# Comparitive Analysis of the Chloroplast Genomes of Three *Houpoea* Plants

**DOI:** 10.3390/genes14061262

**Published:** 2023-06-14

**Authors:** Qinbin Xu, Zhuoran Li, Nannan Wu, Jing Yang, Lang Yuan, Tongxing Zhao, Yongkang Sima, Tao Xu

**Affiliations:** 1 School of Ecology and Environmental Science, Yunnan University, Kunming 650504, China; 2 Kunming Arboretum, Yunnan Academy of Forestry & Grassland Science, Kunming 650201, China

**Keywords:** *Houpoea*, complete chloroplast genome, mutation hot spots, phylogenetic

## Abstract

The genus *Houpoea* belongs to the family Magnoliaceae, and the species in this genus have important medicinal values. However, the investigation of the correlation between the evolution of the genus and its phylogeny has been severely hampered by the unknown range of species within the genus and the paucity of research on its chloroplast genome. Thus, we selected three species of *Houpoea*: *Houpoea officinalis* var *officinalis* (OO), *Houpoea officinalis* var*. biloba* (OB), and *Houpoea rostrata* (R). With lengths of 160,153 bp (OO), 160,011 bp (OB), and 160,070 bp (R), respectively, the whole chloroplast genomes (CPGs) of these three *Houpoea* plants were acquired via Illumina sequencing technology, and the findings were annotated and evaluated. These three chloroplast genomes were revealed by the annotation findings to be typical tetrads. A total of 131, 132, and 120 different genes were annotated. The CPGs of the three species had 52, 47, and 56 repeat sequences, which were primarily found in the ycf2 gene. A useful tool for identifying species is the approximately 170 simple sequence repeats (SSRs) that have been found. The border area of the reverse repetition region (IR) was studied, and it was shown that across the three *Houpoea* plants, it is highly conservative, with only changes between *H. rostrata* and the other two plants observed. Numerous highly variable areas (rps3-rps19, rpl32-trnL, ycf1, ccsA, etc.) have the potential to serve as the barcode label for *Houpoea*, according to an examination of mVISTA and nucleotide diversity (Pi). Phylogenetic relation indicates that *Houpoea* is a monophyletic taxon, and its genus range and systematic position are consistent with the Magnoliaceae system of Sima Yongkang-Lu Shugang, including five species and varieties of *H. officinalis* var. *officinalis*, *H. rostrata*, *H. officinalis var. biloba*, *Houpoea obovate*, and *Houpoea tripetala*, which evolved and differentiated from the ancestors of *Houpoea* to the present *Houpoea* in the above order. This study provides valuable information on the genus *Houpoea*, enriches the CPG information on *Houpoea* genus, and provides genetic resources for the further classification of and phylogenetic research on *Houpoea*.

## 1. Introduction

*Houpoea* was the name given to *Magnolia* sect. *Rytidospermum* Spach by N.H. Xia and C.Y. Wu in 2008 [1]; Sect. *Rytidospermum* was the name given to *Rytidospermum* in the 1996 edition of *Flora Republicae Popularis Sinicae. Houpoea* plants include magnolol, magnocurarine, isomagnolol, and other medicinal components [2]. The dried bark, root, and branch bark of *H. officinalis* var. *officinalis* (Rehder & E.H. Wilson) N.H. Xia & C.Y. Wu or *H. officinalis* var. *biloba* (Rehder & E.H. Wilson) Sima & Hong Yu are designated as “Houpu”, a traditional Chinese medicine (TCM) with valuable therapeutic value [3]. Additionally, they may be employed as afforestation tree species and garden decorative tree species due to their benefits in terms of wood, color, and tree shape [4]. However, because of excessive collection, the natural population of Houpoea species has rapidly declined [5]. In addition, despite extensive study on the pharmacological components and breeding of *Houpoea* species [6,7,8], little is known about their genetic traits. In addition, the species composition and range of *Houpoea* is not clear enough: in the Xia Nianhe System [9,10,11], there are nine species of *Houpoea*, but the Sima Yongkang-Lu Shugang System [12] only includes five species and varieties, while in the Figlar–Nooteboom System [13] and the Wang Yubing System [14], *Houpoea* belongs to the *Mognolia* section *Rytidospermum*, a group of *Houpoea*, and only includes four species.

The chloroplast genome (CPG) is a superior option to the nuclear genome for studying nucleotide diversity and reconstructing the phylogeny of related species because it has obvious advantages over the nuclear genome (a smaller genome size, lower nucleotide substitution rate, parthenogenesis, and haploid characteristics) [15]. The cost of obtaining a genome sequence has significantly decreased as a result of the next-generation sequencing technology’s quick development [16,17,18]. Inferring evolutionary links at a higher taxonomic level is therefore increasingly carried out using data from the chloroplast genome scale, and significant progress has been made even at a lower taxonomic level [19]. Species identification, structural change detection, nucleotide diversity assessment, phylogenetic connection resolution, and evolutionary history reconstruction have all been made possible with the use of CPG comparison and phylogenetic analysis [20]. The complexity of the nuclear genome [21,22,23,24,25] and the physical similarities of the *Houpoea* species make CPG more appropriate for phylogenetic analysis, species identification, and determining *Houpoea* preservation strategies [26].

In order to achieve these goals, we chose three endemic Houpoea taxa from China: *H. officinalis var* officinalis, *H. officinalis var. biloba*, and *H. rostrata* (W.W. Smith) N.H. Xia & C.Y. Wu. Although they are mostly located in diverse regions, including Hunan, Sichuan, and southern Yunnan, and have various growing environments, their chemical and pharmacological components are quite comparable [2,27,28]. As a way to filter out several distinct nucleotide sequences, we examined the CPGs of these three *Houpoea* taxa after obtaining their CPGs using high-throughput Illumina sequencing. The phylogenetic tree was then built to determine the genetic link between the three different taxa of *Houpoea*. These studies will serve as invaluable tools for the categorization of *Houpoea* as well as for the identification of varieties, assessment of quality, and genetic enhancement of *Houpoea*.

## 2. Materials and Methods

### 2.1. Materials and Chloroplast DNA Extraction

*H. rostrata* was gathered from Zhiben Mountain, Caojian Town, Yunlong County (25°45′47.76″ N, 99°5′55.99″ E, 2158 m above sea level), Yunnan Province, China, *H. officinalis* (25°8′59″ N, 102°44′41″ E, 1902 m altitude) and *H. officinalis var. biloba* (25°9′3″ N, 102°44′39″ E, 1910 m altitude) were harvested from Kunming Arboretum, Panlong District, Kunming City, Yunnan Province, China. Fresh and tender leaves were gathered, dried, and stored in the refrigerator at −20 °C. The species was determined by Sima Yongkang.

For operation and extraction, a DNA kit extraction technique was employed [29]. The chloroplast DNA extracted in this study was purified via the high salt–low PH method, and the extracted DNA was detected as follows. First, 2 μL of DNA and 2 μL of 10 × loading buffer were absorbed, labeled with a 2000 bp DNA Maker, and electrophoresis was carried out at 150 V for 25 min. Then, detection was carried out and recorded with a gel imager. Sangon Biotechnology Company (Shanghai, China) used the Illumina platform to carry out the sequencing. Using the TruseqTM RNA sample preparation kit, a DNA library was created. The result was a library with an average insertion size of 400 bp. A total of 4.5 GB of 150 bp paired terminal readings was acquired and saved in FastQ format following library sequencing. The quality of the raw data was regulated with FastQC. After removing the low-quality reading and adapter, assembled the high-quality reader with the latest version of GetOrgenelle. 1.7.4 We applied [30], and then used sequin, version 16.0 [31], for manual correction.

### 2.2. Annotation and Physical Map Drawing

Prokka [32] carried out the gene prediction. The CDD, KOG, COG, NR, and NT databases were compared using NCBI Blast+ [33], GO annotations were obtained using Uniprot, and the KEGG database’s genes were annotated using KAAS [34] (KEGG Automatic Annotation Server). Additionally, it was compared to *Magnolia officinalis*, GenBank number is MW373503, which was included in Geneious 2022.0.1, in the NCBI repository (https://www.ncbi.nlm.nih.gov/ accessed on 31 October 2022) belonging to the authors of [35]. The physical maps were drawn online using CHLOROPLOT (https://irscope.shinyapps.io/Chloroplot/ accessed on 31 October 2022).

### 2.3. SSR and Repeat Sequences Analysis

The minimum repetitive size was chosen to be 30 bp, and REPuter [36] recognized repeat sequences comprising forward, backward, and palindromes with a Hamming distance of 3 bp. The settings were specified as default parameters when using the tandem repeats finder to identify tandem repetitions [37]. MISA detected SSR loci with the following parameters: the repeats of a single nucleotide, dinucleotide, trinucleotide, tetranucleotide, pentanucleotide and hexanucleotide, which are 8, 4, 4, 3, 3, and 3, respectively [38].

### 2.4. Genomic Comparative Analysis

Utilizing the mVISTA program, the complete CPGs of *H. officinalis*, *H. officinalis var. biloba*, and *H. rostrata* were compared. [39]. The Zhang et al. approach yielded variable characters with lengths greater than 200 bp in both coding and non-coding areas. [40]. The calculation of nucleotide variability was carried out using Genepioneer (http://112.86.217.82:9919/#/data?type=3/ accessed on 15 November 2022). The IR\SC border regions of three different plants were examined by the IRSCOPE (https://irscope.shinyapps.io/irapp/ accessed on 15 November 2022) [41].

### 2.5. Phylogenesis Analysis

The three species of *Houpoea*’s CPG sequences were used, and we downloaded 26 other CPG data sets of Magnoliaceae from the NCBI database. The CPGs of these species were aligned using MAFFT [42], Geneious (2022) built the phylogenetic tree via the ML (maximum likelihood approach) approach [35].

## 3. Results

### 3.1. CPG Organizations

**Figure 1:**
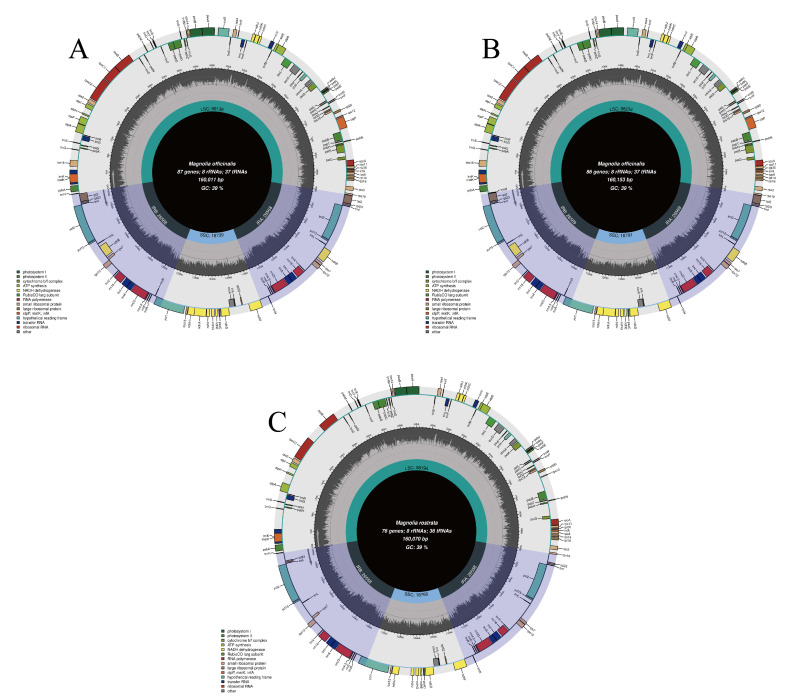
Gene maps of the three *Houpoea* CPGs. (**A**) *H. officinalis var. biloba*; (**B**) *H. officinalis* var. *officinalis*; (**C**) *H. rostrata*. Two IRs, one SSC, and one LSC are the four partition structures displayed. The inner ring is black, designating the GC content, and is marked on the product.

The NCBI database received complete CPGs for the plants *H. officinalis* var. *officinalis*, *H. officinalis var. biloba*, and *H. rostrata* that were acquired via sequencing. Their respective login numbers are OM912809, OM912810, and MW800876. We compared and examined three CPG sequences. As shown in Figure 1, the findings demonstrate that the three CPGs each have a typical four-partition structure and range in size from 160,011 bp (*H. officinalis var. biloba*) to 160,153 bp (*H. rostrata*). The LSC (88,136–88,234) and SSC (18,739–18,781) divide the IRs (26,558–26,569). The GC content of the *H. officinalis* var. *officinalis*, *H. officinalis var. Biloba,* and *H. rostrata* CPGs is 39.26%, 39.22%, and 39.26%, respectively (Table 1), showing similar levels (~39%). The content and sequence of non-protein genes in *Houpoea* CPGs are similar: they all have 8 rRNA genes and 36 or 37 tRNA genes, but the contents and sequences of the protein-coding genes are different: *H. officinalis* var. *officinalis*, *H. officinalis var. biloba*, and *H. rostrata* contain 86, 87, and 76 protein-coding genes, respectively. In other types of genes, *H. rostrata* is also very different from the other two kinds of *Houpoea* plants: clPp and ycf3 are deleted (Table 2).

When the CPGs of three different *Houpoea* plants were compared, it was discovered that six genes had identically sized introns and exons (Table 3).

Among the other differential fragments, the difference between *H. officinalis* var. *officinalis* and *var. biloba* is mainly in the class I intron (InI, the length difference is within 3 bp). Compared with the first two kinds of *Houpoea*, *H. rostrata* shows great differences, and introns and exons are only detected in rps12 and a few tRNA genes. The above results also show that there is little difference between *H. officinalis* var. *Officinalis* and *var. biloba*, and their CPGs are richer in structure and function than those of *H. rostrata*.

### 3.2. Analysis of the Repeat and the SSRs

**Figure 2:**
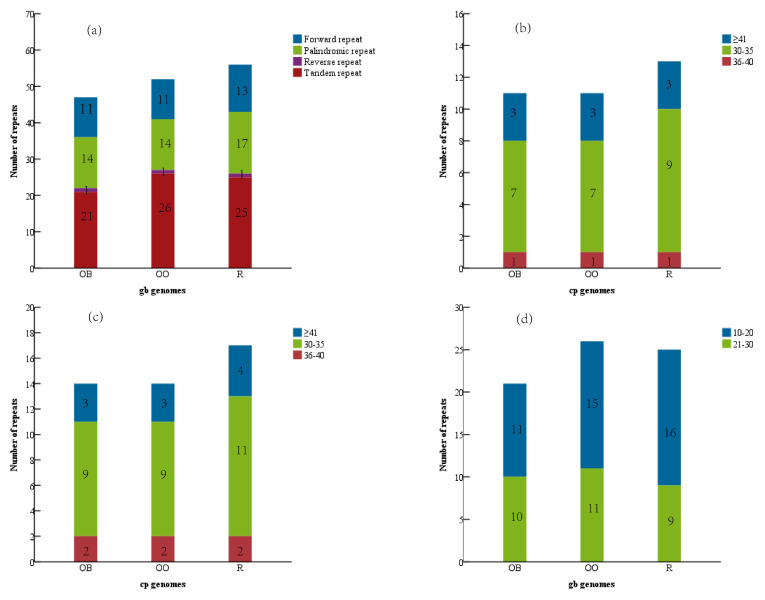
Examination of three CPGs’ repeated sequences. (**a**) repetition style; (**b**) forward repetition; (**c**) palindrome repetitions; (**d**) tandem repetition. In (**a**), various colors depict various sorts; in (**b**–**d**), various colors denote various lengths. The ordinate stands for quantity, and the abscissa for three CPGs.

The examination of the repeated sequences in *Houpoea* CPGs reveals that the distribution and quantity of the four different types of repetitive sequences (tandem, forward, reverse, and palindrome repetitive sequences) are comparable (Figure 2a). *H. officinalis* var. *officinalis* has the most tandem repetitions among them (26), whereas *H. officinalis* var*. biloba* has the fewest tandem repeats (21). Although *H. rostrata* (13, 17) has the most forward and palindrome repetitions compared to the other two (11, 14), the number of reverse repeats is the same (1). The three repetitions all follow the same pattern in terms of length: the tandem repeats are often 10–20 bp long (Figure 2d), but the forward and palindrome repeats are typically 30–40 bp long (Figure 2b,c). In addition, the repeat sequences of the three *Houpoea* plants are mainly distributed in the ycf2 gene and intergenic regions such as ycf3-ycf19 (Table S1 Repeat sequences of chloroplast genome of Three *Houpoea officinalis*).

**Figure 3:**
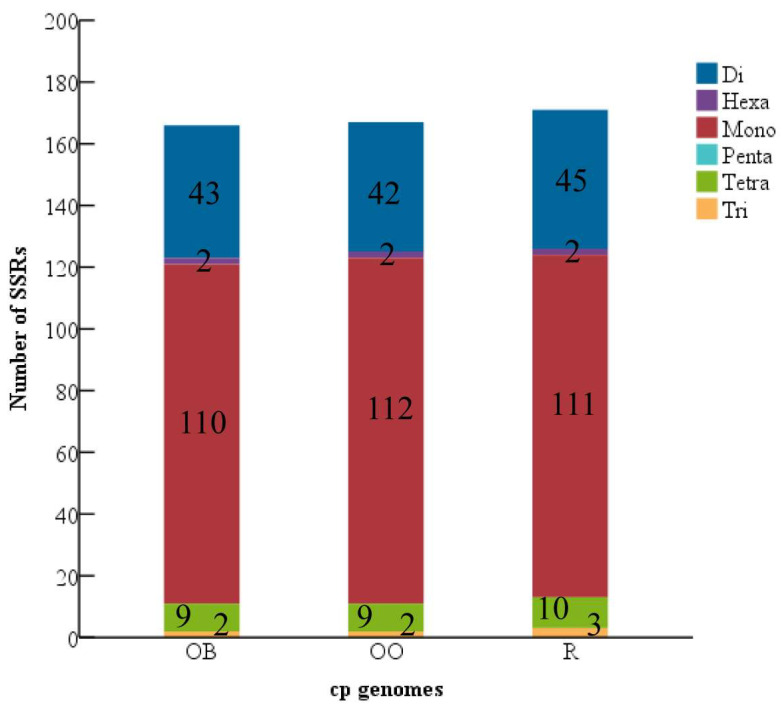
SSR research on three *Houpoea*’s CPGs. The abscissa represents the CPGs of three *Houpoea*, whereas the ordinate reflects the number of SSRs.

The SSRs are very variable within a single species, but the CPGs are monogenic [42,43]. They serve as molecular markers in developmental studies and aid in species identification as a result [44]. MISA detected 166 SSR markers in *H. officinalis var. biloba*, including 110 single nucleotides, 43 dinucleotides, 2 trinucleotides, 9 tetranucleotides, and 2 hexanucleotide repeats. Additionally, 167 SSRs and 171 SSRs, which include 112—111 single nucleotides, 42—45 dinucleotides, 2—3 trinucleotides, 9—10 tetranucleotides, and 2 hexanucleotide repeats, were found in the CPGs of *H. officinalis* and *H. rostrata*, respectively. No pentanucleotide duplication was discovered either (Figure 3).

### 3.3. Comparative Genomics

**Figure 4:**
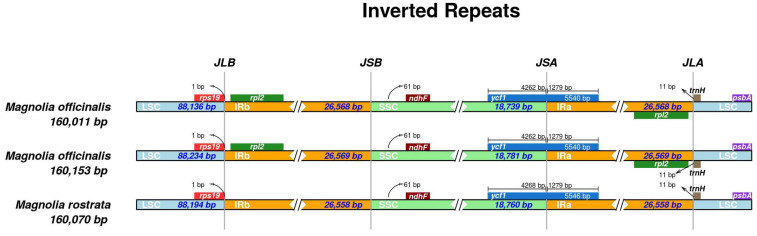
Comparison of the three *Houpoea* species’ chloroplast genome border regions (LSC, SSC, and IRs).

The IR regions are the most conservative areas in the CPGs in most angiosperms. The length of an IR region is inversely proportional to the genome’s length. [44]. In order to evaluate and study the IR, LSC, and SSC border regions of these three *Houpoea* species, the findings revealed that the IR region’s length varied between 26,558 and 26,569 BP (Table 1), and while variances across the three species were minimal, the IR border region did exhibit some noticeable disparities. Figure 4 illustrates how *H. rostrata* deleted the rpl2 gene in comparison to the other two in the IRs/LSC border areas. This demonstrates that the *H. rostrata* CPGs have a more conservative structure and function.

**Figure 5:**
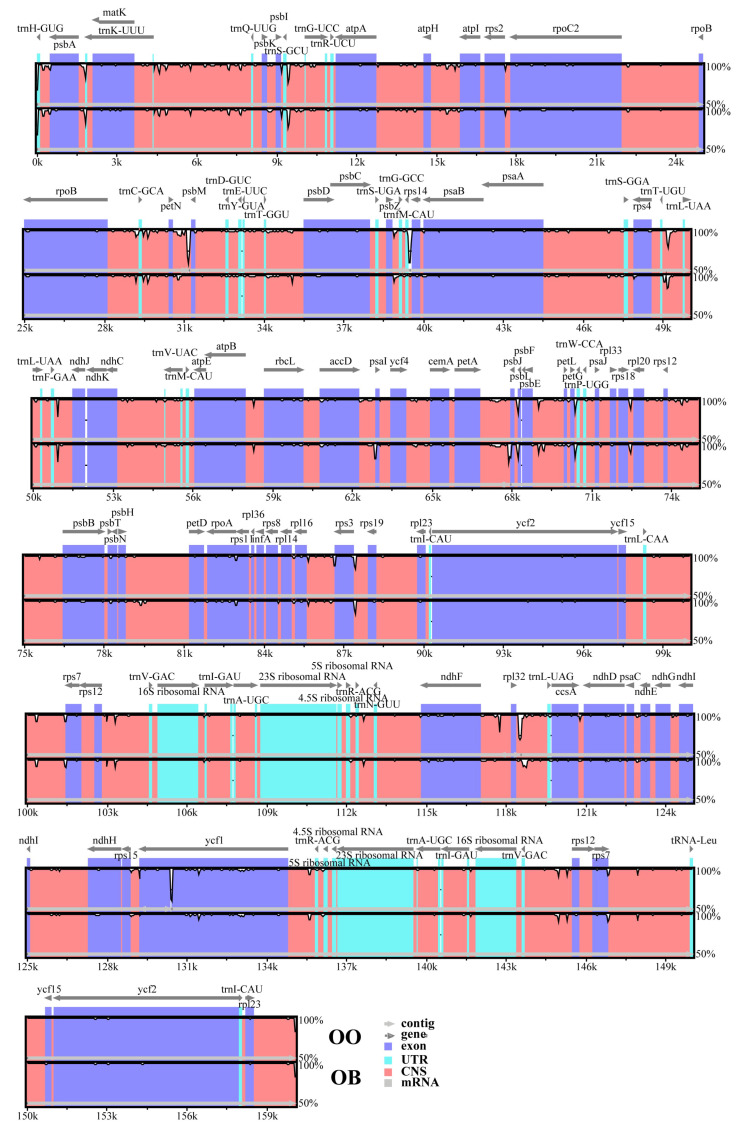
The CPG sequences of three *Houpoea* chloroplasts were compared using mVISTA based on *H. rostrata*. The arrow shows the direction of transcription, and the colors are used to identify distinct areas of the CPG.

In the mVISTA comparison of three CPGs using H. rostrata as a reference, three Houpoea CPGs have a significant degree of sequence conservation, as seen in Figure 5, particularly in the coding area. Compared to gene areas, intergenic regions have mutation sites that are easier to find. The most highly variable areas are in the psbM-petN, trnfM-rps14, trnL-trnT, and psbL-psbJ, etc., which are the conserved non-coding sequence (CNS) regions.

**Figure 6:**
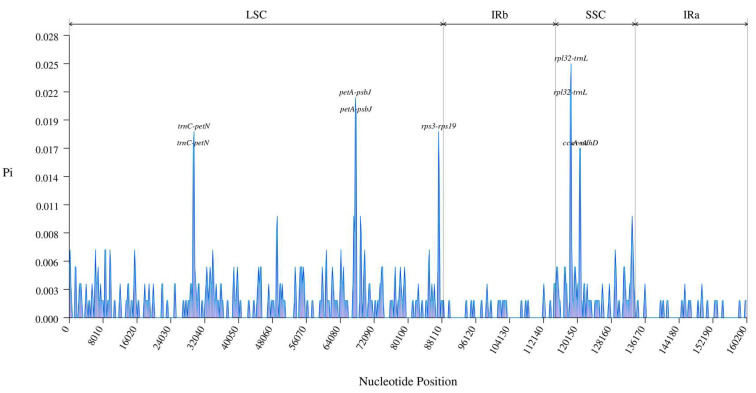
Comparing *H. officinalis*, *H. officinalis var. biloba*, and *H. rostrata*’s nucleotide variability (Pi) values.

The nucleotide diversity value (Pi) was determined using Genepioneer in order to intuitively explain the high variation region (Figure 6). According to the findings, the percentage range is 0 to 2.5%, with an average of 0.16%. Ten areas with a Pi greater than 1% were discovered, which were various places for nine intergenic regions (rpl32-trnL, petA-psbJ, rpl32-trnL, petA-psbJ, trnC-petN, rps3-rps19, trnC-petN, ccsA-ndhD, and rps3-rps19) and one gene (trnL). The pl32-trnL has the highest Pi value (2.5%). Additionally, only the LSC and SSC areas include these highly variable regions, which is consistent with the structural flexibility of CPGs.

### 3.4. Phylogenetic Analysis

**Figure 7:**
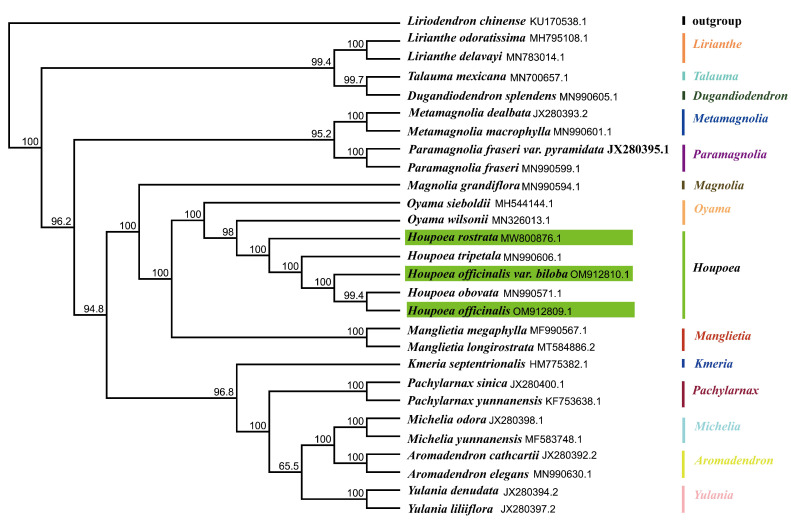
Based on the entire sequence of 29 CPGs, a maximum likelihood tree was created. The species name and the sequence’s GenBank login number are indicated in the illustration. A number for boot support based on 1000 copies is displayed next to the node. Each genus is labeled according to Sima-Lu’s system and distinguished by color.

It has been established that the plastid offers more phylogenetic signals than fragment DNA markers, which is crucial for establishing the deep relationships in plant ancestry [45]. To examine the genetic connections between *H. officialis* var. *officinalis*, *H. officialis var. biloba*, and *H. rostrata* in more detail, *Liriodendron Chinense* (Hemsley) Sargent was used as the outer group, and 25 CPG sequences from the Magnoliaceae family were chosen. The maximum likelihood approach was used to build the phylogenetic tree. As seen in Figure 7, *Houpoea obovata* and *H. officinalis* var. *officinalis* were clustered together. This branch was then clustered with *H. officinalis var. biloba* and *H. tripetala* (Linnaeus) Sima, S.G. Lu, N.H. Xia & C.Y. Wu in turn, all of which had a 100% support rate. Meanwhile, *H. rostrata* is at the base of the genus *Houpoea* branch, formed sister group with the other three branches, and also had a 100% support rate. This phylogenetic analysis also provides a reference for the species composition and range of *Houpoea*. In the Sima -Lu’s System, *Houpoea* includes five species and varieties which are also reflected in the ML tree of this study, while the Xia Nianhe system has a wider range, including four groups: *Paramagnolia fraseri var. fraser*i (Walter) Sima & S. G. Lu, *P. fraseri var. pyramidata* (Bartram) Sima & S. G. Lu, *Metamagnolia macrophylla* (Michaux) Sima & S. G. Lu, and *Met. dealbata* (Zuccarini) Sima & S. G. Lu. In this study, these four groups are clustered into another branch far from the branch of *Houpoea*. It can be seen that the Xia’s system definition of *Houpoea* is a polyphyletic group.

### 3.5. Plant Morphology

Table 4 shows that the physical traits of the flowers and fruits are identical, while the color of the leaf coat and the form of the apex are the key variations between the three species of *Houpoea* plants. The morphological characteristics of *H. rostrata* are the most different from those of the other two plants, among which the leaves of *H. rostrata* are reddish brown with long hair, wide and round apexes, and are short and acute. However, *H. officinalis* var. *biloba* and var. *officinalis* have long white leaves and short sharp or obtuse tips. The difference between them is that the apex of an *H. officinalis* var. *biloba* leaf is concave and forms two obtuse, shallow lobes.

## 4. Discussion

High-quality DNA sample preparation is the first step in genome sequencing research. Compared with the nuclear genomic DNA, the extraction of chloroplast genomic DNA is more difficult, and the complete chloroplast must be obtained first. In the process of chloroplast DNA separation, there may be problems such as nuclear DNA, mitochondrial DNA pollution, and chloroplast integrity, which are the key factors restricting chloroplast DNA extraction. To obtain the high-quality chloroplast genomic DNA required for this research, chloroplast DNA was isolated in this work, purified via the high salt–low PH approach [46] to eliminate interfering DNA, and identified via gel electrophoresis.

Most angiosperms have CPGs that are between 120 and 160 kb in size [47]. The findings demonstrate that the CPGs of the three species of *Houpoea* are comparable in extent (approximately 160 kb) and composition (tetrad structure) to other Magnoliaceae plants [48] and other higher plants [49]. However, there are differences in the types and quantities of genes, among which *H. rostrata* (120) is the most different from the other two species (131-132). We hypothesize that *H. officinalis* var. *bilob*a and var. *officinalis* have given rise to more variants throughout the course of evolution, while *H. rostrata*, the most ape-like species of *Houpoea* [50], has a more ape-like and conservative chloroplast genome. In particular, the GC content is greater in early differentiated lineages such as the Magnoliaceae. It has been shown that the total GC content is connected to the phylogenetic position [51]. Our findings agree with earlier research. The overall GC concentration of CPGs in the three species of *Houpoea* plants is roughly 39.2%, which is greater than the median GC content of most angiosperms (35%) [52].

Due to their structural variety in various CPG areas and their crucial involvement in plastid recombination, repeated sequences are frequently employed in phylogeny, population genetic analysis, and evolutionary studies [53]. The interspersed repeat sequence and tandem repeat sequence were detected in three species of *Houpoea*. All three types of Houpoea species lack complement repeats, which are the most common kind of interspersed repeat sequence. The difference in CPGs is caused by various ratios of forward, palindrome, and reverse repetitions [54]. The results of this study conform to this model: *H. officinalis var. biloba* (11, 14, 1) and var. *officinalis* (11, 14, 1) are very different from *H. rostrata* (13, 17, 1). Previous research has demonstrated that intergenic spacer regions, followed by coding areas, are where repeat sequences are most commonly found [55]. Our study demonstrates that genes such as ycf2 and intergenic spacer regions are primarily associated with the repetitive sequences of the three *Houpoea* plants (Table S1). The distribution of repeat sequences in CPGs is a significant source of structural diversity and a key factor in genome evolution [56]. As a result, the ycf2 gene, which contains a significant number of repeat sequences, may be the key to understanding the variations between the three types of *Houpoea*.

SSRs are molecular markers with significant application potential that have been extensively utilized in phylogenetic research, species breeding and protection, species identification, and other domains [57]. H. rostrata carries the most SSR markers among the three species of Houpoea, and single nucleotide repeats predominate in all of them. Pentanucleotide repeats are absent in all three CPGs, which is typical with other species of Magnoliaceae, and tetranucleotide repeats are often somewhat more prevalent than trinucleotide repeats and hexanucleotide repeats [58,59,60]. A strong foundation for related research, such as species identification, is provided by the SSRs of the CPGs of the three *Houpoea* species discovered in this work.

Through a comparison and analysis of the full CPG sequences of the three *Houpoea*, we discovered that the differences between the SSC and LSC regions are larger than those in the IR area, and the area that does not code is larger than the programming region. This finding was in line with previous studies on other Magnoliaceae plants [61]. This study shows that there are obvious differences among the three species in the IR boundary region: when comparing *H. officinalis* var. *biloba* and var. *officinalis*, *H. rostrata*, with a long beak, has lost the rpl2 gene at the IRb-LSC region. Our best opinion is that during evolution, changes in the living environment caused the Houpoea species to evolve. Moreover, through mVISTA comparison, we identified 10 regions showing significant differences in the CPGs of these three plants: nine areas with intergenic spacers (psbA-trnK, trnS-trnG, psbM-petN, trnfM-rps14, trnL-trnT, trnF-ndhJ, psbL-psbJ, rps3-rps19, and rpl32-trnL) and one gene (ycf1). Numerous evolutionary details are provided by the highly variable areas, which may also be employed as possible molecular markers to distinguish between related taxa [62]. Many highly variable areas, such as matk and ycf1, have been exploited to create DNA barcodes in Magnoliaceae. [63]. Nucleotide diversity (Pi) and mVISTA allowed us to identify ten highly variable areas, such as one gene (ccsA) and nine areas with intergenic spacers (rpl32-trnL, petA-psbJ, trnC-petN, rps3-rps19, trnC-petN, and CCSA). These regions may serve as *Houpoea* species identification barcode labels.

In resolving the evolutionary connection between angiosperms, the CPGs have demonstrated tremendous power [64]. *Houpoea* species’ distributions are unclear [11]; hence, further proof is required to confirm their range. Consequently, the CPG sequences of 29 groupings of Magnoliaceae plants were used to create a phylogenetic tree via ML. The findings demonstrate a monophyly among all *Houpoea* sequences which is compatible with the classification of *Houpoea* under the Sima -Lu’s System [12]. Among them, *H. rostrata* is located at the base of *Houpoea*, and then *H. officinalis* *var. biloba*, *H. officinalis* var. *officinalis*, *H. obovata*, and *H. tripetala* are separated in turn. It also shows the evolution of *Houpoea*: the species of *Houpoea* originated in the Himalayas, spread northeast to Central China, evolved into *H. officinalis var. Biloba*, and differentiated into *H. officinalis* var. *officinalis*. Through the Bering Strait, ocean currents, and birds, it migrated to the eastern and southern regions of North America at the same time as it continued to travel northeast to Japan, where it differentiated into *H. obovata*.

## 5. Conclusions

In this work, the CPGs of three species of *Houpoea* plants (*H. officinalis var. biloba*, *H. officinalis* and *H. rostrata*) were sequenced, annotated, and compared. The findings indicate that these three distinct species’ CPGs are very conservative in terms of structure and gene content, but they also exhibit distinct variances that reflect their genetic link. A total of 170 SSR loci were also discovered as molecular markers to investigate the variety of *Houpoea*. The DNA barcode of the *Houpoea* species may consist of 10 extremely variable regions (rps3-rps19, rpl32-trnL, ycf1, ccsA, etc.). The division of *Houpoea* by the Sima Yongkang-Lu Shugang System is supported by the phylogenetic relationship, which demonstrates the clear evolutionary background of *Houpoea* and the grouping of all species of *Houpoea* into a single line. All the aforementioned information improved the genus *Houpoea*’s genome data and offers valuable resources for future studies on the phylogeny and species identification of the genus.

## Figures and Tables

**Table 1:** Basic information of CPGs of three *Houpoea* plants.

Lable	Species	Genome Size (bp)	LSC Region (bp)	IR Region (bp)	SSC Region (bp)	Number of Genes	PCGs	tRNAs	rRNAs	GC Content (%)	Number of Accession in Genbank
OB	*H. officinalis* *var. biloba*	160,011	88,136	26,568	18,739	132	87	37	8	39.22	OM912810
R	*H. rostrata*	160,070	88,194	26,558	18,760	120	76	36	8	39.26	MW800876
OO	*H. officinalis*	160,153	88,234	26,569	18,781	131	86	37	8	39.26	OM912809

**Table 2:** Gene list in the CPGs of three *Houpoea* plants.

Category	Gene Group	Gene Name
Photosynthesis	Photosystem I	psaA, psaB, psaC, psaI, psaJ
	Photosystem II	psbA, psbB, psbC, psbD, psbE, psbF, psbH, psbI, psbJ, psbK, psbL, psbM, psbN, psbT, psbZ
	NADH dehydrogenase	ndhC, ndhD, ndhE, ndhF, ndhG, ndhH, ndhI, ndhJ, ndhK, ndhA*, ndhB(2), ndhA*, ndhB(2)
	Cytochrome b/f complex	petA, petD, petG, petL, petN, petB*, petB*
	ATP synthase	atpA, atpB, atpE, atpH, atpI, atpF*, atpF*
	Large subunit of rubisco	rbcL
Self-replication	Proteins ribosomal (LSU)	rpl14, rpl16, rpl20, rpl23(2), rpl32, rpl33, rpl36, rpl2*(2), rpl2*(2), rpl32
	Proteins ribosomal (SSU)	rps11, rps12**(2), rps14, rps15, rps18, rps19, rps2, rps3, rps4, rps7(2), rps8, rps16*, rps16*
	RNA polymerase	rpoA, rpoB, rpoC2, rpoC1*, rpoC1*
	Ribosomal RNAs	rrn16(2), rrn23(2), rrn4.5(2), rrn5(2)
	Transfer RNAs	tRNA-Leu, trnA-UGC*(2), trnC-GCA, trnD-GUC, trnE-UUC, trnF-GAA, trnG-GCC, trnG-UCC*, trnH-GUG, trnI-CAU(2), trnI-GAU*(2), trnK-UUU*, trnL-CAA, trnL-UAA*, trnL-UAG, trnM-CAU, trnN-GUU, trnP-UGG, trnQ-UUG, trnR-ACG(2), trnR-UCU, trnS-GCU, trnS-GGA, trnS-UGA, trnT-GGU, trnT-UGU, trnV-GAC(2), trnV-UAC*, trnW-CCA, trnY-GUA, trnfM-CAU, trnA*, trnI, trnI*, trnI-CAU, trnI-GAU*, trnL, trnL*trnM, trnV, trnV-GAC, trnA-UGC*, trnD, trnA-UGC*(2), trnI-CAU(2), trnI-GAU*(2), trnL, trnL-UAA*, trnN-GUU(2), trnV-GAC(2), trnfM-CAU
Other genes	Maturase	matK
	Protease	clpP** clpP**
	Envelope membrane	cemA
	Acetyl-CoA carboxylase	accD
	c-type cytochrome synthesis gene	ccsA
	Translation initiation factor	infA
	Conserved hypothetical chloroplast ORF	ycf1, ycf15(2), ycf2(2), ycf4, ycf3**, ycf3**

Notes: Gene*: one-intron-containing gene; Gene**: two-intron gene; Gene(2): the number of multi-copy genes; The colors of gene names represent different species: genes-R, genes-OO, and genes-OB.

**Table 3:** Comparison of introns and exons of the CPGs.

Gene	OO					OB					R				
	EpI	InI	EpII	InII	EpIII	EpI	InI	EpII	InII	EpIII	EpI	InI	EpII	InII	EpIII
rps12 ^a^	114	536	232		26	114	536	232		26	114	536	232		26
trnK-UUU	37	2491	35			37	2491	35			37	2491	35		
rps16	42	824	219			42	824	219							
trnG-UCC	24	770	48			24	768	748			24	766	48		
atpF	144	711	411			144	709	411							
rpoC1	434	722	1624			434	722	1624							
ycf3	126	734	228	728	153	126	734	228	728	153					
trnL	35	491	50												
trnL-UAA						35	491	50			35	491	50		
trnV-UAC	39	584	37			39	584	37			39	584	37		
rps12 ^b^	114	536	232		26	114	536	232		26	114	536	232		26
clpP	69	785	291	628	246	69	782	291	629	246					
petB	6	784	642			6	779	642							
rpl2 ^a^	393	658	432			393	658	432							
ndhB ^a^	777	700	756			777	700	756							
trnI-GAU ^a^	42	936	35			42	936	35			42	936	35		
trnA-UGC ^a^	38	799	35			38	799	35			38	799	35		
ndhA	552	1080	540			552	1078	540							
trnA	38	799	35												
trnI	42	936	35												
trnA-UGC ^b^						38	799	35			38	799	35		
trnI-GAU ^b^						42	936	35			42	936	35		
ndhB ^b^	777	700	756			777	700	756							
rpl2 ^b^	393	658	432			393	658	432							

Note: ^a^ IRA region; ^b^ IRB region.

**Table 4:** The comparison of Botanical traits of three *Houpoea* plants.

Botanical Traits	OO	OB	R
Leaf	White, long hair, concave leaf apex, shallow lobes in two obtuse circles.	White, long hair, short, acute or obtuse at the apex of leaves, without notch.	White, reddish-brown hair, apex broadly rounded, with a short, acute point.
Fruit	Aggregate fruit; oblong ovoid	Aggregate fruit; oblong ovoid.	Aggregate fruit is cylindrical and upright.
Flower	White, fragrant, 12 cm in diameter.	White, fragrant, 13 cm in diameter.	White, fragrant, 9 cm in diameter.

Note: OO-*H. officinalis* var. *officinalis*; OB-*H. officinalis* var. *biloba*; R-*H. rostrata*.

## Data Availability

The chloroplast genome data mentioned in this study can be obtained from GenBank of NCBI at https://www.ncbi.nlm.nih.gov.

## Supplementary Materials

The following supporting information can be downloaded at: https://www.mdpi.com/article/10.3390/genes14061262/s1.
