# Supplementary material for: Comparitive Analysis of the Chloroplast Genomes of Three Houpoea Plants
Source: Genes (Basel). 2023 Jun 14;14(6):1262. doi: 10.3390/genes14061262 (PMC10298425; doi:10.3390/genes14061262)
Supplement: Supplementary file 1 [file genes-14-01262-s001.zip › genes-2430115-supplementary.pdf]

Table S1 Repeat sequences of chloroplast genome of Three Houpoea officinalis

Interspersed repeats					Tandern repeats				
ID	Length(bp)	Type	Location		ID	Length(bp)	Copy Number	Location	
H.officinalis var. biloba									
1	42	F	ycf2	IRA	1	17	2.0	ycf3	LSC
2	42	P	ycf2	IRA	2	15	2.1	rps16-trnQ	LSC
3	42	P	ycf2	IRA	3	23	2.0	trnI-GAU	LSC
4	42	F	ycf2	IRB	4	24	2.0	trnI-GAU	LSC
5	42	P	ycf1	SSC	5	17	2.1	psbM-trnD	LSC
6	40	R	rps3-rps19	LSC	6	20	2.0	trnF-ndhJ	LSC
7	33	F	ycf2	IRA	7	15	2.3	accD-psaI	LSC
8	33	P	ycf2	IRA	8	21	2.1	rps18	LSC
9	33	P	ycf2	IRA	9	15	3.3	rpl20-rps12	LSC
10	33	F	ycf2	IRB	10	24	2.0	rps11	LSC
11	39	F	ycf3	LSC	11	21	2.1	rps3-rps19	LSC
12	39	P	ycf3	LSC	12	15	2.1	ycf2	IRA
13	41	F	psaB	LSC	13	21	4.1	ycf2	IRA
14	31	F	ycf2	IRB	14	24	2.4	ycf2	IRA
15	32	P	petA-psbJ	LSC	15	18	3.4	trnN-ndhF	IRA
16	37	P	rps15-ycf1	SSC	16	18	2.2	ycf1	SSC
17	30	P	trnS-GCU	LSC	17	18	2.2	ycf1	SSC
18	31	F	trnS-GCU	LSC	18	18	3.4	ycf1-trnN	SSC
19	33	P	psbM-trnD	LSC	19	24	2.4	ycf2	IRB
20	30	F	trnN-ndhF	IRA	20	21	4.1	ycf2	IRB
21	30	P	trnN-ndhF	IRA	21	15	2.1	ycf2	IRB
22	30	P	trnN-ndhF	IRA					
23	30	F	ndhF	IRB					
24	30	P	trnS-UGA	LSC					
25	30	F	ycf3	LSC					
26	30	P	ycf3	LSC					
H.officinalis									
1	42	F	ycf2	IRA	1	17	2.0	psbA-trnK	LSC
2	42	P	ycf2	IRA	2	15	2.1	rps16-trnQ	LSC
3	42	P	ycf2	IRA	3	23	2.0	trnl	LSC
4	42	F	ycf2	IRB	4	24	2.0	trnl	LSC
5	42	P	ycf1	SSC	5	17	2.1	psbM-trnD	LSC
6	40	R	rps3-rps19	LSC	6	26	2.0	trnM-rps14	LSC
7	33	F	ycf2	IRA	7	20	2.0	trnF-ndhJ	LSC
8	33	P	ycf2	IRA	8	15	2.3	hgdskfjl	LSC
9	33	P	ycf2	IRA	9	17	2.1	accD-psaI	LSC
10	33	F	ycf2	IRB	10	21	2.1	rps18	LSC
11	39	F	ycf3	LSC	11	15	3.3	rpl20-rps12	LSC
12	39	P	ycf3	LSC	12	24	2.0	rps11	LSC
13	41	F	psaB	LSC	13	14	1.9	rps16-rps3	LSC

Interspersed repeats					Tandern repeats				
ID	Length(bp)	Type	Location		ID	Length(bp)	Copy Number	Location	
14	31	F	ycf2	IRB	14	21	2.1	rps3-rps19	LSC
15	32	P	petA-psbJ	LSC	15	15	2.1	ycf2	IRA
16	37	P	rps15-ycf1	SSC	16	21	4.1	ycf2	IRA
17	30	P	trnS-GCU	LSC	17	24	2.4	ycf2	IRA
18	31	F	trnS-GCU	LSC	18	18	3.4	trnN-ndhF	IRA
19	33	P	psbM-trnD	LSC	19	22	2.1	ndhF-trnL	SSC
20	30	F	trnN-ndhF	IRA	20	11	2.6	rps15-ycf1	SSC
21	30	P	trnN-ndhF	IRA	21	18	2.2	ycf1	SSC
22	30	P	trnN-ndhF	IRA	22	18	2.2	ycf1	SSC
23	30	F	ndhF	IRB	23	18	3.4	ycf1-trnD	SSC
24	30	P	trnS-UGA	LSC	24	24	2.4	ycf2	IRB
25	30	F	ycf3	LSC	25	21	4.1	ycf2	IRB
26	30	P	ycf3	LSC	26	15	2.1	ycf2	IRB
H. rostrata									
1	42	F	ycf2	LSC	1	15	2.1	trnK-trnQ	LSC
2	42	P	ycf2	LSC	2	22	2.4	trnS-trnG	LSC
3	42	P	ycf2	LSC	3	22	2.0	atpA-atpH	LSC
4	42	F	ycf2	IRB	4	17	2.1	psbM-trnD	LSC
5	44	P	atpH-atpI	LSC	5	16	2.1	trnT-trnL	LSC
6	42	P	ycf1	SSC	6	15	2.0	trnT-trnL	LSC
7	33	F	ycf2	IRA	7	20	2.0	trnF-ndhJ	LSC
8	33	P	ycf2	IRA	8	15	2.3	accD-psaI	LSC
9	33	P	ycf2	IRA	9	17	2.1	accD-psaI	LSC
10	33	F	ycf2	IRB	10	17	2.0	psbJ-psbL	LSC
11	39	F	psaA-trnS	LSC	11	12	2.2	petG-trnW	LSC
12	39	P	psaA-trnS	LSC	12	21	2.1	rps18	LSC
13	41	F	psaB	LSC	13	15	3.3	rpl29-rps12	LSC
14	38	R	rps3-rps19	LSC	14	24	2.0	rps11	LSC
15	35	P	rps2-rpoC2	LSC	15	21	2.1	rps3-rps19	LSC
16	31	F	ycf2	IRB	16	15	2.1	ycf2	IRA
17	32	P	petA-psbJ	LSC	17	21	4.1	ycf2	IRA
18	37	P	rps15-ycf1	SSC	18	24	2.4	ycf2	IRA
19	30	P	trnS-GCU	LSC	19	18	3.4	trnN-ndhF	IRA
20	30	F	trnS-trnG	LSC	20	18	2.2	ycf1	SSC
21	31	F	trnS-GCU	LSC	21	18	2.2	ycf1	SSC
22	33	P	rps2-rpoC2	LSC	22	18	3.4	ycf1-trnR	SSC
23	33	P	psbM-trnD	LSC	23	24	2.4	ycf2	IRB
24	30	F	trnN-ndhF	IRA	24	21	4.1	ycf2	IRB
25	30	P	trnN-ndhF	IRA	25	15	2.1	ycf2	IRB
26	30	P	trnN-ndhF	IRA					
27	30	F	ycf1-trnR	SSC					
28	31	F	rps2-rpoC2	LSC					
29	30	P	trnS-UGA	LSC					

Interspersed repeats					Tandern repeats			
ID	Length(bp)	Type	Location		ID	Length(bp)	Copy Number	Location
30	30	F	psaA-trnS	LSC				
31	30	P	psaA-trnS	LSC				

Note：P：palindromic repeat，F：forward repeat，R：reverse repeat

Table S2 Complete chloroplast genome sequencing species of Magnoliaceae

ID	Genebank	Name of NCBI	Latin name
1	MN783014	*Magnolia delavayi*	*Lirianthe delavayi* (Franchet) N. H. Xia & C. Y. Wu
2	MH795108	*Magnolia odoratissima*	*Lirianthe odoratissima* (Y. W. Law & R. Z. Zhou) N. H. Xia & C. Y. Wu
3	MH544144	*Magnolia sieboldii*	*Oyama sieboldii* (K. Koch) N. H. Xia & C. Y. Wu
4	MN326013	*Magnolia wilsonii*	*Oyama wilsonii* (Finet & Gagnepain) N.H.Xia & C.Y.Wu
5	MN990594	*Magnolia grandiflora*	*Magnolia grandiflora* Linnaeus
6	MN990599	*Magnolia fraseri*	*Paramagnolia fraseri* var. *fraseri* (Walt er) Sima & S. G. Lu
7	JX280395.1	*Magnolia pyramidata*	*Paramagnolia fraseri var. pyramidata* (Bartram) Sima & S. G. Lu
8	NC037001	*Magnolia conifera*	*Manglietia conifera* Dandy
9	MT584886.2	*Magnolia longirostrata*	*Manglietia longirostrata* (D. X. Li & R. Z. Zhou ex X. M. Hu, Q. W. Zeng & L. Fu) Sima
10	MF990567.1	*Magnolia dandyi*	*Manglietia megaphylla* Hu & W. C. C heng
11	KF753638.1	*Magnolia yunnanensis*	*Pachylarnax yunnanensis* (Hu) Sima & S. G. Lu
12	JX280400.1	*Magnolia sinica*	*Pachylarnax sinica* (Y. W. Law) N. H. Xia & C. Y. Wu
13	HM775382.1	*Magnolia kwangsiensis*	*Kmeria septentrionalis* Dandy
14	MF583748.1	*Michelia yunnanensis*	*Michelia yunnanensis* Franchet ex Finet & Gagnepain
15	JX280398.1	*Michelia odora*	*Michelia odora* (Chun) Nooteboom & B. L. Chen
16	JX280392.2	*Magnolia cathcartii*	*Aromadendron cathcartii* (J. D. Hooker & Thomson) Sima & S. G. Lu
17	MN990630.1	*Magnolia elegans*	*Aromadendron elegans* Blume
18	JX280394	*Magnolia denudata*	*Yulania denudata* (Desrousseaux) D. L. Fu
19	JX280397	*Magnolia liliiflora*	*Yulania liliiflora* (Desrousseaux) D. L. Fu
20	MN700657.1	*Magnolia mexicana*	*Talauma mexicana* (Candolle) G. Don
21	MN990605.1	*Magnolia splendens*	*Dugandiodendron splendens* (Urban) Si ma & S. G. Lu
22	KU170538	*Liriodendron chinense*	*Liriodendron chinense* (Hemsley) Sarge nt
23	MW800876	*Magnolia rostrata*	*Houpoea rostrata* (W. W. Smith) N. H. Xia & C. Y. Wu

24	OM912809	*Magnolia officinalis*	*Houpoea officinalis* var. *officinalis* (Rehder & E. H. Wilson) N. H. Xia & C. Y. Wu
25	OM912810	*Magnolia officinalis subsp. biloba*	*Houpoea officinalis* var. *biloba* (Rehder & E. H. Wilson) Sima & Hong Yu
26	MN990571	*Magnolia obovata*	*Houpoea obovata* (Thunberg) N. H. Xia & C. Y. Wu
27	MN990606.1	*Magnolia tripetala*	*Houpoea tripetala* (Linnaeus) Sima, S. G. Lu, N. H. Xia & C. Y. Wu
28	MN990601.1	*Magnolia macrophylla*	*Metamagnolia macrophylla* (Michaux) Sima & S. G. Lu
29	JX280393.2	*Magnolia macrophylla var. dealbata*	*Metamagnolia dealbata* (Zuccarini) Sima & S. G. Lu
